# Salidroside Protects against Cadmium-Induced Hepatotoxicity in Rats via GJIC and MAPK Pathways

**DOI:** 10.1371/journal.pone.0129788

**Published:** 2015-06-12

**Authors:** Hui Zou, Xuezhong Liu, Tao Han, Di Hu, Yi Wang, Yan Yuan, Jianhong Gu, Jianchun Bian, Jiaqiao Zhu, Zong-ping Liu

**Affiliations:** College of Veterinary Medicine, Yangzhou University, and Jiangsu Co-innovation Center for Prevention and Control of Important Animal Infectious Diseases and Zoonoses, Yangzhou, Jiangsu, P.R. China

## Abstract

It is known that cadmium (Cd) induces cytotoxicity in hepatocytes; however, the underlying mechanism is unclear. Here, we studied the molecular mechanisms of Cd-induced hepatotoxicity in rat liver cells (BRL 3A) and in vivo. We observed that Cd treatment was associated with a time- and concentration-dependent decrease in the cell index (CI) of BRL 3A cells and cellular organelle ultrastructure injury in the rat liver. Meanwhile, Cd treatment resulted in the inhibition of gap junction intercellular communication (GJIC) and activation of mitogen-activated protein kinase (MAPK) pathways. Gap junction blocker 18-β-glycyrrhetinic acid (GA), administered in combination with Cd, exacerbated cytotoxic injury in BRL 3A cells; however, GA had a protective effect on healthy cells co-cultured with Cd-exposed cells in a co-culture system. Cd-induced cytotoxic injury could be attenuated by co-treatment with an extracellular signal-regulated kinase (ERK) inhibitor (U0126) and a p38 inhibitor (SB202190) but was not affected by co-treatment with a c-Jun N-terminal kinase (JNK) inhibitor (SP600125). These results indicate that ERK and p38 play critical roles in Cd-induced hepatotoxicity and mediate the function of gap junctions. Moreover, MAPKs induce changes in GJIC by controlling connexin gene expression, while GJIC has little effect on the Cd-induced activation of MAPK pathways. Collectively, our study has identified a possible mechanistic pathway of Cd-induced hepatotoxicity in vitro and in vivo, and identified the participation of GJIC and MAPK-mediated pathways in Cd-induced hepatotoxicity. Furthermore, we have shown that salidroside may be a functional chemopreventative agent that ameliorates the negative effects of Cd via GJIC and MAPK pathways.

## Introduction

Cadmium (Cd) is a serious environmental toxicant with harmful effects on health in both animals and humans. It is known to target multiple organ systems, particularly the kidneys and liver[1]. Damage resulting from Cd-induced oxidative stress activates signaling cascades, including the Ca^2+^ pathway, the mitogen-activated protein kinase (MAPK) pathway, the phosphatidylinositol-3-kinase (PI3K)-Akt pathway and the nuclear factor-κB (NF-κB) pathway, which cause cellular injury, apoptosis and carcinogenesis[2]. However, the definitive signaling pathway that plays the crucial role in Cd-induced apoptosis remains unclear.

Gap junction intercellular communication (GJIC) is one of the most important cellular communications and plays an important role in many biological processes [3, 4]. Gap junctions are formed from two connexons on adjacent cells, with each connexon comprised of six connexins (Cx). GJIC, by nature, implies the passive diffusion of small (<1000 Da), hydrophilic substances (e.g., ions, small metabolites and secondary messengers) and there are more than 20 connexin species known to be present in animals and humans, where Cx32 makes up about 90% of the hepatic connexin content. Connexin gene expression and gap junction channel gating are two major mechanisms of GJIC control [5]. It is well known that the functional loss of gap junctions can result in apoptosis, necrosis and carcinogenesis [6–9] and it is also known that Cd disrupts gap junction activity in hepatocytes in vitro and in vivo [10, 11].

MAPKs are a family of Ser/Thr protein kinases of highly conserved enzymes that are unique to eukaryotes. They have been shown to participate in many facets of cellular regulation, such as the control of gene expression, cell proliferation and programmed cell death [12]. Extracellular signal-regulated kinase (ERK), JNK and p38 MAPK are three major members of the MAPK family. These enzymes are activated by phosphorylation and the strength and duration of the activated MAPKs, as well as the cell type, determine the diversity of gene expression, which results in different physiological consequences. Studies have shown that MAPKs are involved in Cd-induced apoptosis in various cell types, including hepatocytes [13].

Salidroside (Sal) is an active constituent of *Rhodiola rosea* L., which has been used over many years as a medicinal herb for the treatment of altitude sickness [14]. Previous studies have shown that Sal exhibits several pharmacological activities, including anti-oxidant, anti-aging, anti-inflammatory, anti-cancer, anti-fatigue and anti-depressant effects [15–19]. Additional studies have found that Sal exerts a protective effect against cellular injury and apoptosis by altering signal transduction in cells. For example, Sal has been shown to protect brain neurons from ischemic injury via the mammalian target of rapamycin (mTOR) signaling pathway [20] and has also been shown to protect against oxygen–glucose deprivation (OGD)/re-oxygenation-induced H9c2 cell necrosis via activation of Akt-Nrf2 signaling [21]. Furthermore, Sal has been found to attenuate H_2_O_2_-induced bone marrow-endothelial progenitor cell (BM-EPC) apoptosis by inhibiting the up-regulation of phosphorylated c-Jun N-terminal kinase (JNK) and p38 MAPK, whilst down-regulating the Bax/Bcl-xL expression ratio [22].

Based on these considerations, in this study we chose Sal as a protective agent against Cd-induced apoptosis to investigate the interactional effects of the MAPK pathway and GJIC, as well as the protective mechanism of Sal both in vitro and in vivo.

## Materials and Methods

### Materials

Sal (purity >99%) was obtained from the National Institute for the Control of Pharmaceutical and Biological Products (Beijing, China). Dulbecco’s modified Eagle’s medium (DMEM) and fetal bovine serum (FBS) were obtained from Gibco (Grand Island, NY, USA). The enhanced chemiluminescence (ECL) detection kit was from Millipore (Burlington, MA, USA). Cadmium acetate (Cd), Lucifer yellow (LY), 18-β-glycyrrhetinic acid (GA), U0126, SP600125, SB202190 and the anti-ERK, anti-P-ERK, anti-JNK, anti-P-JNK, anti-p38, anti-P-p38 and anti-β-actin antibodies were purchased from Sigma–Aldrich (Shanghai, China). PrimeScript RT Reagent kit and SYBR Premix Ex Taq were obtained from TaKaRa Biotechnology (Dalian, China). Oligonucleotide primers were synthesized by Invitrogen (Shanghai, China). All other reagents were of analytical grade.

### Animals and treatment

Thirty female Sprague-Dawley rats weighing 80–100 g were obtained from the Laboratory Animal Center of Jiangsu University (Zhenjiang, China). The animals were housed individually on a 12 h light/dark cycle with unlimited standard rat food and double distilled water (DDW). All experimental procedures were conducted in accordance with the recommendations in the Guide for the Care and Use of Laboratory Animals of the National Research Council and were approved by the Animal Care and Use Committee of Yangzhou University (Approval ID: SYXK (Su) 2007–0005). All surgeries operations were performed under sodium pentobarbital anesthesia, and all efforts were made to minimize any suffering experienced by the animals used in this study. The animals were divided randomly into three groups as follows. (1) Control group: 10 rats consuming DDW as their drinking water. (2) Cadmium group: 10 rats consuming a solution of Cd (50 mg/L) as their drinking water. (3) Cadmium + Sal group: 10 rats treated daily with Sal (35 mg/kg body weight, intragastric gavage, i.g.) and consuming a solution of Cd (50 mg/L) as their drinking water. All rats were sacrificed by cervical dislocation 12 weeks after initial treatment.

### Cell culture

Rat liver cells (BRL 3A) were purchased from the Cell Bank of the Institute of Biochemistry and Cell Biology (Shanghai, China). The cells were suspended in DMEM supplemented with 10% FBS, 100 U/mL penicillin and 100 μg/mL streptomycin, and then incubated in a humidified 5% CO_2_/95% air atmosphere at 37°C. BRL 3A cells from passages 10 to 30 were used for all experiments and treated at 90% confluence with different concentrations of Cd (2.5 μM or 10 μM) in the presence or absence of Sal (50 μM) for 12 h.

### Real time analysis of cytotoxicity using an xCELLigence DP system

The xCELLigence system consists of three components: an analyzer, a real-time cell analyzer (RCTA) station and an E-plate. The dimensionless parameter cell index (CI) was used to quantify cell status and was derived from the measured cell–electrode impedance that directly relates to cell number, cell viability, adhesion and morphology[23].

The xCELLigence system (Roche Applied Science, Basel, Switzerland) was operated according to the manufacturer’s instructions [24]. The background impedance of the E-plate was determined in 100 mL medium. Then, 100 mL of the BRL 3A cell suspension was added (10,000 cells/well). Cells were incubated for 30 min in the incubator and then the E-plate was placed into the xCELLigence station. The CI was measured every 15 min. During the phase of rapid CI increase, the growth medium was replaced by serum-free culture medium containing different compounds according to the experimental design. The results were normalized at the end of the assay.

### Western blot analysis

Equal amounts of protein (40 μg), were separated by 10% sodium dodecyl sulfate-polyacrylamide gel electrophoresis and transferred onto nitrocellulose membranes. The membrane was incubated with 5% nonfat milk in Tris-buffered saline with 0.1% Tween-20 (TBST) at room temperature for 30 min before incubation with primary antibodies against ERK, P-ERK, JNK, P-JNK, p38, P-p38 (1:1000) or β-actin (1:5000) overnight at 4°C, followed by incubation with horseradish peroxidase (HRP)-conjugated goat anti-rabbit IgG (1:5000) at room temperature for 2 h. The membranes were then washed with TBST and the protein bands were visualized by ECL reagents. The results were analyzed using Image Lab software (Bio-Rad, Hercules, CA, USA).

### Scrape-loading/dye-transfer assay for GJIC

A scrape-loading/dye-transfer method (SL/DT) for assessing GJIC using LY was performed as originally described by El-Fouly et al. [25]. In brief, after washing three times with PBS, the cells or fresh liver tissue were scraped with a sharp blade in the presence of LY (0.5 mg/mL), followed by incubation in the dark for 3 min at 37°C and then washed three times with PBS and fixed with 4% paraformaldehyde. The liver tissue samples were then processed by a standard histological technique and mounted on glass slides. The level of GJIC was quantified as the average distance traveled by the LY dye from the scraped edge to the neighboring cells, which was measured using a fluorescent microscope (Leica DMI 3000B, Solms, Germany).

### Transmission electron microscopy and immuno-electron microscopy

Internal cellular structures were examined using a Philips CM-100 transmission electronic microscope. Fresh liver tissue was cut into small pieces and fixed with glutaraldehyde (2.5% in 0.1 mol/L cacodylate buffer, pH 7.4) at 4°C for 24 h and embedded in agar. Then, the samples were treated with 1% osmium tetroxide for 2 h. After dehydration with a graded acetone series, the samples were placed in pure acetone and Epon 812 epoxy solution for 30 min and then embedded in Epon 812. Ultrathin sections were cut with a diamond knife and stained with uranyl acetate and lead citrate for transmission electron microscopy. For immuno-electron microscopy, before staining with uranyl acetate and lead citrate, the sections were incubated with Cx32 antibody (1:100) at 4°C overnight, then incubated with 5 nm colloidal gold-conjugated anti-rabbit IgG (Sigma–Aldrich, Shanghai, China; dilution: 1:100) at 4°C for 4 h.

### RNA extraction, reverse transcription and quantitative reverse transcription polymerase chain reaction (qRT-PCR)

Total RNA was extracted from cultured cells and liver tissue using TRIzol Reagent (Invitrogen) according to the manufacturer’s instructions. The cDNA was synthesized from 900 ng total RNA using a PrimeScript RT Reagent kit with gDNA Eraser (Takara, Japan). The primers were designed using Primer Premier 5 as follows: β-actin forward, 5'- CGTTGACATCCGTAAAGACCTC-3' and reverse, 5'- TAGGAGCCAGGGCAGTAATCT -3'; Cx32 forward, 5'- TGGAAGAGGTAAAGAGGCACAAG-3' and reverse, 5'- GGCGGGACACGAAGCAGT-3'. The expression levels of all genes were measured using a real-time PCR system (Applied Biosystems 7500, USA) and the reactions were performed with a SYBR Premix Taq II kit (Takara, Japan) according the manufacturer’s instructions. mRNA levels were analyzed with the ΔΔ*C*_T_ method.

### Transwell culture of BRL 3A cells

**Fig 1:**
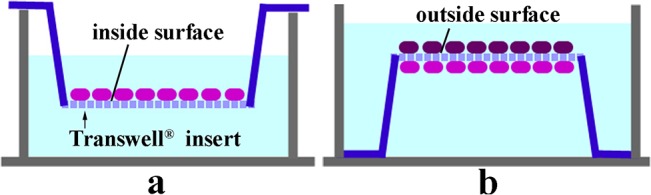
Transwell culture system. BRL 3A cells were cultured on the inside surfaces of Transwell cell culture inserts in serum-free DMEM with 10 μM Cd for 12 h (a). The inserts were then overturned and BRL 3A cells seeded on the outside surface of the inserts(b), with both sides then incubated in culture medium with 50 μM Sal, 5 μM GA, 10 μM U0126, 10 μM SP600125 or 10 μM SB202190 for 36 h.

BRL 3A cells were seeded at a density of 5×10^4^ cells/cm^2^ on the inside surface of the polyester membrane of Transwell cell culture inserts (pore size 0.4 μm, surface area 4.67 cm^2^; Corning, UK), then incubated in serum-free DMEM with 10 μM Cd for 12 h after confluence was achieved. After that, the insert was overturned and BRL 3A cells seeded at a density of 5×10^4^ cells/cm^2^ on the outside surface of the insert before incubation of the insert in culture medium with 50 μM Sal, 5 μM GA, 10 μM U0126, 10 μM SP600125 or 10 μM SB202190 for 36 h (Fig 1). Finally, cells were carefully wiped from the inside surface of the insert with a cotton swab and the insert washed with cold PBS three times. Cells were fixed in 4% (*w*/*v*) paraformaldehyde (Sigma–Aldrich, Shanghai, China) in PBS for 15 min and then stained for 15 min with Hoechst 33258. Images were obtained by an inverted fluorescence microscope (Leica, Germany).

### Statistical analysis

All of the experimental data are presented as the mean ± standard deviation (S.D.). Statistical data comparisons among groups were performed using a non-parametric, one way analysis of variance (ANOVA) with *p <* 0.05 considered statistically significant.

## Results

### Cd triggers cytotoxicity in BRL 3A cells and rat liver tissue

**Fig 2:**
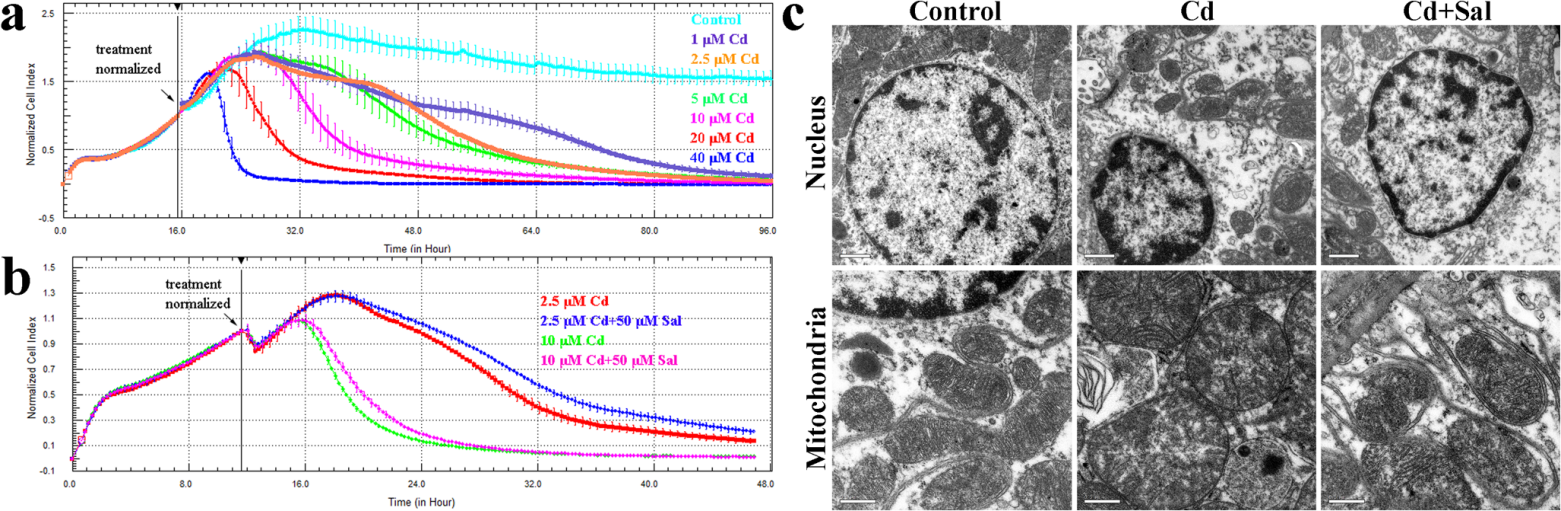
Cd triggers cytotoxicity in BRL 3A cells and rat liver tissue. **a**, **b**: The effect of Cd treatment and Sal on the cell index in BRL 3A cells. Data were normalized at the time of treatment. Curves were plotted as an average of quadruplicate treatments. Error bars show the standard deviation. **c**: Transmission electron microscopy reveals the ultrastructure changes of nuclei and mitochondria. Bar = 0.5 μm.

We evaluated Cd toxicity in BRL 3A cells using the xCELLigence DP system. We found that treatment with Cd (1, 2.5, 5, 10, 20 and 40 μM) resulted in a time- and concentration-dependent decrease of the cell index (CI) in BRL 3A cells (Fig 2A). To assess the influence of Sal on Cd-induced cytotoxicity, cells incubated in the presence of Sal were compared to cells treated with Cd alone. In comparison to BRL 3A cells exposed to Cd alone, the addition of Sal was associated with a slower decrease of the CI (Fig 2B). These results show that Cd triggers cytotoxic injury in a time and dose-dependent manner and Sal at a concentration of 50 μM, can ameliorate the effect of Cd.

Transmission electron microscopy was used to confirm that the mitochondria and the nuclei were indeed affected by Cd (Fig 2C). In untreated cells the nuclei were normal, with evenly distributed chromatin, and the mitochondria did not exhibit edema and had clear mitochondrial cristae. Cd was noted to induce nuclei shrinkage and chromatin karyopyknosis, as evidence by the intense staining and marginalization. Mitochondrial swelling was noted and mitochondrial cristae fused partly and became blurry or were even missing. These cellular organelle ultrastructure injuries were partly rescued by treatment with Sal.

### GJIC has a dual effect on Cd-induced cytotoxic injury

**Fig 3:**
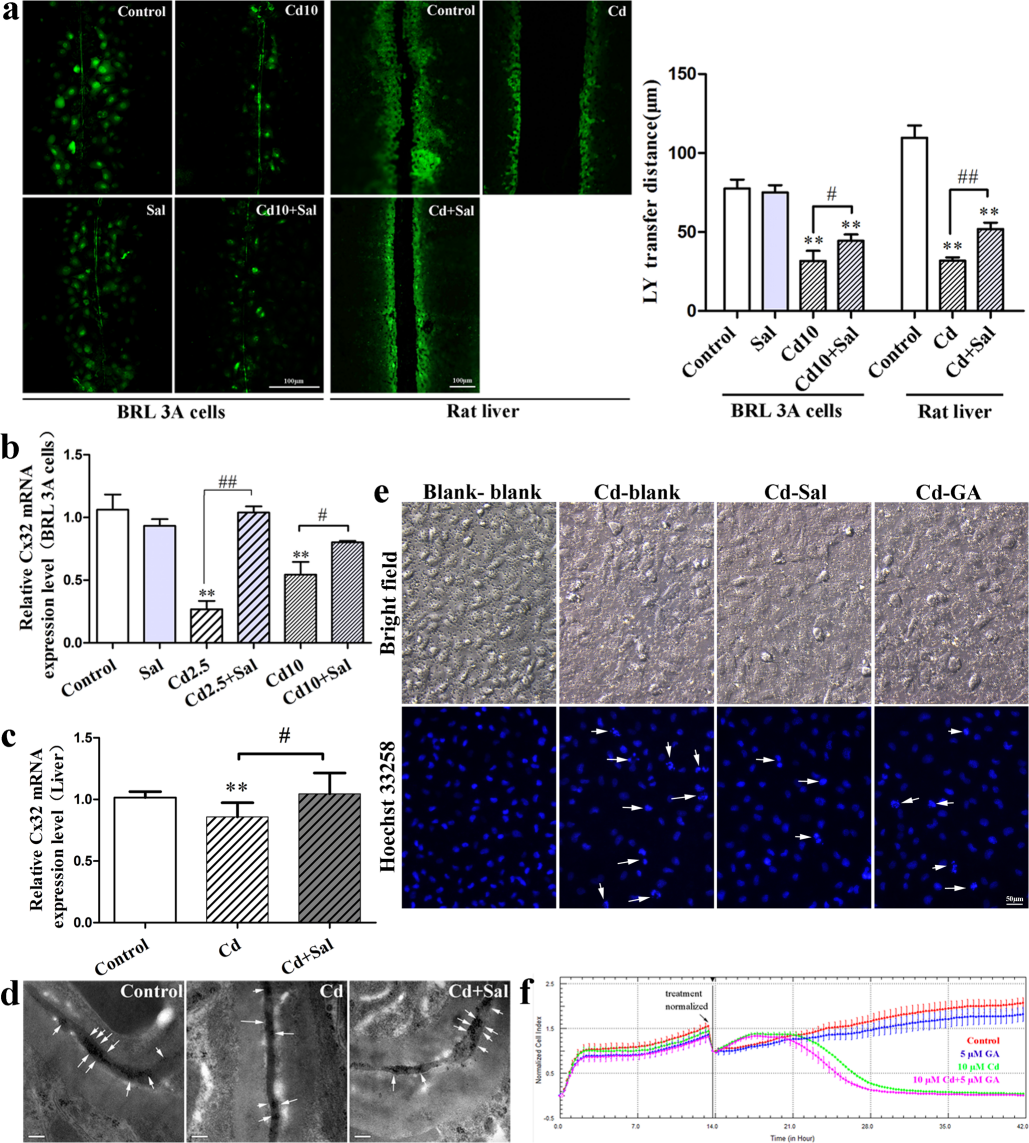
GJIC has a dual effect on Cd-induced cytotoxic injury. **a**: Effect of Sal on the Cd-induced down-regulation of GJIC in BRL 3A cells and rat liver tissue(Cd 2.5 means 2.5μm Cd, Cd 10 means 10μm Cd). LY transferred to adjacent cells via open gap junctions. Scale bar = 100 μm. The average distance of LY spread from the side of the scraped edge from six different sites in each sample was obtained for quantification; **b**, **c**: Effect of Sal on Cd-induced decreases in Cx32 mRNA(relative Cx32 mRNA expression in BRL 3A cells and rat liver tissue). Values are the means ± S.D. ** *p* < 0.01 compared with the control group, # *p* < 0.05, ## *p* < 0.01 compared with the group indicated. **d**: Effect of Sal on Cd-induced alterations in the distribution of Cx32 in rat hepatocytes. Gold particles indicating Cx32 were observed at closely apposed plasma membranes of the hepatocytes, as shown by the arrows. Gold particle size = 5 nm. Scale bar = 0.2 μm. **e**: BRL 3A cells *influenced by* Cd-exposed cells in a Transwell culture system. Cells were divided between the two sides of the insert according to their treatment. Scale bar = 50 μm. **f**: Effect of 5 μM GA on 10 μM Cd-induced cytotoxicity in BRL 3A cells.

To determine gap junction activity after treatment of BRL 3A cells and rat liver tissue with Cd, the GJIC was measured by a scrape-loading/dye-transfer method. We found that treatment with Cd resulted in inhibition of GJIC in both BRL 3A cells and rat liver tissue, while Sal had a protective effect on GJIC (Fig 3A). We then investigated the effect of Cd treatment on the mRNA expression level of Cx32 using qRT-PCR. As shown in Fig 3B and Fig 3C, the relative expression level of Cx32 was significantly (*p* < 0.05 or *p* < 0.01) decreased following Cd treatment compared with the control group, while co-treatment with Sal significantly attenuated the Cd-induced decrease of Cx32 mRNA. Immuno-labeled Cx32 was observed by transmission electron microscopy (Fig 3D). In the control group, Cx32 was seen at the gap junctions between adjacent hepatocytes; however, the gold particles appeared decreased in number and scattered after Cd treatment. Notably, Sal appeared to suppress the Cd-induced Cx32 mRNA decrease.

Using a Transwell culture system we co-cultured Cd-exposed cells on the inside surface with BRL 3A cells on the outside surface in the presence of 50 μM Sal and 5 μM GA (Fig 3E). Cd-exposed cells exhibited decreased viability, nuclei chromatin condensation and even nuclear fragmentation. However, these injuries could be ameliorated by the addition of 50 μM Sal. To verify the role of GJIC in Cd cytotoxicity, BRL 3A cells were co-treated with 10 μM Cd and 5 μM GA, which is a prototypical gap junction blocker. As shown in Fig 3F, Cd decreased cell viability and co-treatment with GA exacerbated the reduction in cell viability, as well as nuclei injury, compared with the cells treated only with Cd. However, in the Transwell co-culture system, GA had a protective effect on BRL 3A cells co-cultured with Cd-exposed cells (Fig 3E).

### Cd activates the MAPK pathway in BRL 3A cells and rat liver tissue

**Fig 4:**
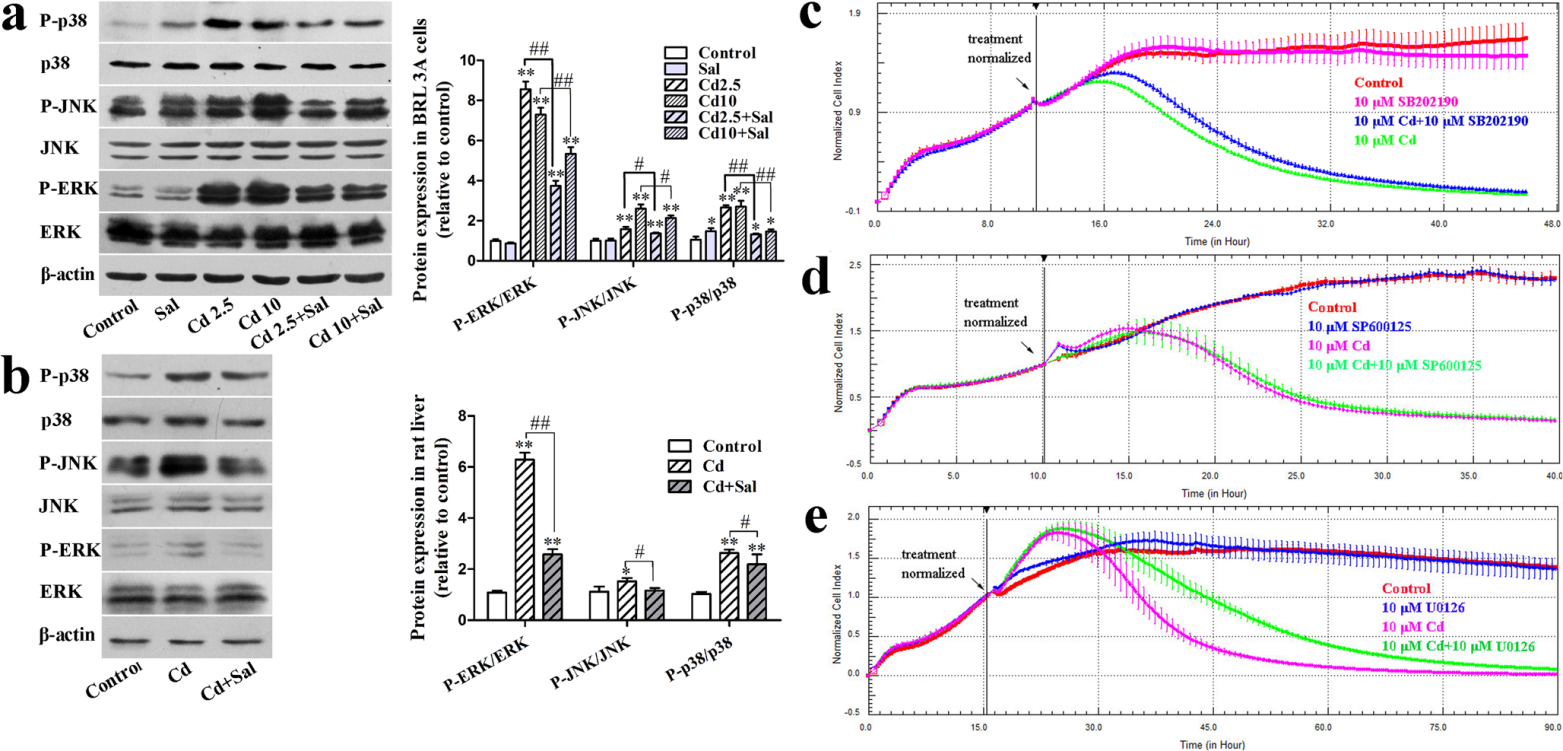
Cd activates the MAPK pathway in BRL 3A cells and rat liver tissue. Effect of Sal on Cd-induced phosphorylation of MAPKs in BRL 3A cells (**a**) and rat liver tissue (**b**). Values shown are the means ± S.D. of at least three independent experiments. Each value is expressed as the phospho/total MAPK percentage of phosphorylation. ** *p* < 0.01 and * *p* < 0.05 compared with control group; # *p* < 0.05 and ## *p* < 0.01 compared with the indicated group. **c**, **d**, **e**: Effects of U0126, SP600125 and SB202190 on Cd-induced cytotoxicity.

Western blot analysis was performed to determine the phosphorylation of MAPKs in BRL 3A cells and rat liver tissue (Fig 4A and 4B). The phosphorylation levels of ERK, JNK and p38 in BRL 3A cells were increased after Cd treatment for 12 h, significantly so for ERK. However, Sal co-treatment significantly inhibited the up-regulation of phosphorylated ERK, JNK and p38. Similar results were also seen in rat liver tissue. To define the roles of MAPKs in Cd-induced cytotoxicity in hepatocytes, cells were co-treated with 10 μM Cd and U0126 (10 μM), SP600125 (10 μM) or SB202190 (10 μM). The ERK inhibito (U0126) and the p38 inhibitor (SB202190) prevented the CI decrease induced by Cd (Fig 4C and 4E). However, the JNK inhibitor (SP600125) had little effect on the decrease of the CI induced by Cd(Fig 4B). These data demonstrate that the phosphorylation of ERK and p38 is essential for Cd-induced cytotoxicity and that co-treatment with Sal has a protective effect.

### The interaction between GJIC and the MAPK pathway plays an important role in Cd-induced cytotoxicity

**Fig 5:**
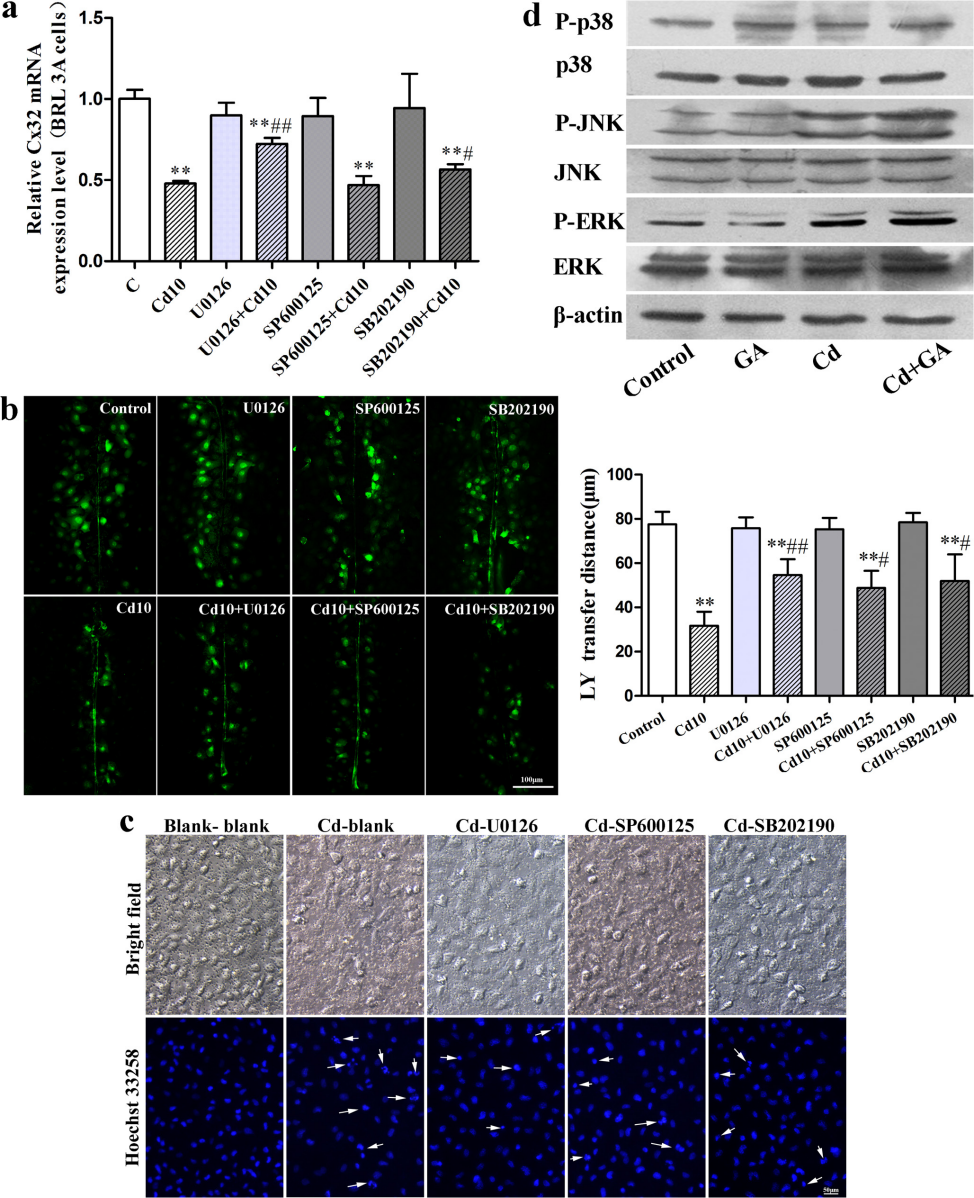
The interaction between GJIC and the MAPK pathway plays an important role in Cd-induced cytotoxicity. **a**: Effect of U0126, SP600125 and SB202190 on Cx32 mRNA expression in BRL 3A cells; **b**: Effect of U0126, SP600125 and SB202190 on the Cd-induced down-regulation of GJIC in BRL 3A cells. LY transferred to adjacent cells via open gap junctions. Scale bar = 100 μm. ** *p* < 0.01 compared with control group; # *p* < 0.05 and ## *p* < 0.01 compared with the 10 μM Cd group. **c**: The influence of Cd-exposed cells on BRL 3A cells in a Transwell culture system. Scale bar = 50 μm. **d**: Effect of GA on the Cd-induced phosphorylation of MAPKs in BRL 3A cells.

To assess the interaction between GJIC and the MAPK pathway following Cd-induced cytotoxicity, MAPK inhibitors (U0126, SP600125 and SB202190) were added to cells in vitro. We found that co-treatment with U0126 significantly blocked decreases in Cd-induced Cx32 mRNA and SB202190 also restored Cx32 levels to some extent, whereas SP600125 had no effect on the expression of Cx32 mRNA (Fig 5A). In addition, alterations in Cx32 mRNA expression were found to be in accordance with GJIC function (Fig 5B). To verify the role of the MAPK pathway in Cd-induced cytotoxicity, BRL 3A cells were incubated in the presence of Cd (10 μM) on the inside surface of a Transwell insert for 12 h (an untreated control was also established). After this time, cells were seeded on the outside surface of the insert in the presence of U0126 (10 μM), SP600125 (10 μM) or SB202190 (10 μM) and compared with untreated control cells and cells treated only with Cd. As shown in Fig 5C, U0126 and SB202190 had a protective effect on Cd-exposed cells, with a decrease in nuclei chromatin condensation and nuclear fragmentation, while SP600125 had little effect. Meanwhile, Fig 5D demonstrates that GA had no effect on the Cd-induced up-regulation of phosphorylated ERK, JNK and p38.

## Discussion

Cd is a persistent environmental contaminant with toxic effects in both humans and animals. There is growing evidence that Cd induces apoptosis, but the underlying mechanism remains unclear. The goal of this study was to examine the toxic mechanism of Cd exposure in rat hepatocytes in vitro and in vivo, with a focus on the MAPK pathways and GJIC. In the present study, Cd was demonstrated to be toxic to rat hepatocytes, resulting in decreased cell viability and inhibition of GJIC function. Furthermore, MAPK pathways were found to have critical functions in Cd-exposed hepatocytes, and GJIC inhibition had the dual effect of protecting healthy cells, while damaging injured cells. The protective agent Sal partly attenuated Cd-induced hepatotoxicity.

Sal, as an efficacious anti-oxidant, has been widely researched both in vitro and in vivo. A previous study has demonstrated that Sal (10 μM, 50 μM and 100 μM) inhibits 1-methyl-4-phenylpyridinium (MPP+)-induced apoptosis in PC12 cells in a concentration-dependent manner [26]. In addition, Sal (20 or 50 mg/kg) has been shown to reduce neuronal damage in C57BL/6 mice [27]. Based on these reports, we selected Sal at a concentration of 50 μM for our in vitro studies and 35 mg/kg body weight (i.g.), for our in vivo studies. According to our findings, Cd exerted a cytotoxic effect in BRL 3A cells in a time- and concentration-dependent manner (Fig 2A), with ultrastructural damage of nuclei and mitochondria in vivo (Fig 2C). Similar findings have been reported in PC12 cells and rat liver tissue [28–30]. These injuries were attenuated by co-treatment with Sal.

In multicellular organisms, the global interplay between the extra-, intra- and inter-cellular communication controls the maintenance of homeostatic balance [31–33]. Direct intercellular communication is mainly mediated by gap junctions [34] and the liver was one of the first organs in which GJIC was studied [35]. In the adult liver, gap junctions occupy about 3% of the hepatocyte membrane surface and Cx32 is the major connexin, comprising as much as 90% of the total connexin content [36]. Previous studies have indicated that GJIC can spatially extend apoptosis through the communication of cell death signals from apoptotic cells to healthy cells [8]. In the present study, Cd induced inhibition of GJIC and down-regulation of Cx32 mRNA expression both in BRL 3A cells and the rat liver (Fig 3A, 3B and 3C). Correspondingly, Cx32 decreased and scattered following Cd treatment (Fig 3D). Meanwhile, GJIC inhibition also caused injured cells to lose the rescue signals (such as glucose, ATP and ascorbic acid) provided by healthy cells, which shows a loss of normal growth regulation by the surrounding cells and growth independence [34]. Cells co-treated with GA, a gap junction blocker, exacerbated the effect of Cd in BRL 3A cells (Fig 3F), which is consistent with previous findings, while GJIC inhibition protected healthy cells by limiting the flux of toxic metabolites, such as nitric oxide and superoxide ions, from conjoint damaged cells [37].

To assess whether healthy cells were affected by apoptosis cells, Guo *et al*. [38] co-cultured normal PC12 cells and Pb^2+^-exposed PC12 cells, which were transfected with the EF1A-eGFP vector. They found that Pb^2+^-exposed PC12 cells induced apoptosis in the unexposed cells via a reactive oxygen species (ROS)-dependent mitochondrial pathway, which was achieved by GJIC. Accordingly, in the present study, Transwell inserts with a 0.4 μm pore size were used in a co-culture system. The pore size was selected so that cells could not cross through the insert membrane but small-size substances could be shared via hemi-channels and/or establishment of gap junctions (as is known for pore sizes less than 3 μm). As such, we co-cultured Cd-exposed BRL 3A cells and normal BRL 3A cells independently on the two sides of a Transwell insert (Fig 1). The resulting findings showed that the inside surface of Cd-exposed cells induced cell damage to the unexposed cells on the outside surface, as shown by nuclei injuries, and that GA and Sal have a protective effect (Fig 3E). This shows that GJIC inhibition has the dual effect of protecting normal cells and exacerbating damage in Cd-exposed cells.

The liver has been identified as a major target of Cd-mediated toxicity; however, not all aspects of the mechanism have been fully elucidated [39]. Cd is known to induce cytotoxic injury via various cell signal-transduction pathways. Wang et al. [40] demonstrated that Cd induced apoptosis via oxidative stress and calcium overload in rat hepatocytes, while Xu et al. [41] have demonstrated that Cd resulted in the caspase-dependent apoptosis of neuronal cells, which was induced by [Ca^2+^]_i_ elevation, implicating ROS and activated MAPK and mTOR pathways. MAPKs are important signaling enzymes that play a critical role in controlling gene expression, cell survival and cell death; however, the regulation of cytotoxic injury by MAPKs is complex [12]. In this study, we showed that ERK, JNK and p38 activation may be involved in Cd-induced hepatotoxicity both in vitro and in vivo (Fig 4A and 4B), which is in agreement with our previous study [42]. Others studies have shown that ERK is mainly activated by growth factors and tumor promoters and is necesssary for cell proliferation and differentiation, whereas JNK and p38 are involved in apoptosis by promoting cell death [43, 44]. Both survival and death signals can activate ERK. Depending on the cell type and stimulus, ERK has a dual effect: besides its involvement in cell proliferation and differentiation, the duration of the activation of ERK also acts as a negative regulator of cell survival and promotes apoptosis [45–47]. Our results showed that ERK, JNK and p38 phosphorylation are altered in Cd-exposed BRL 3A cells and the injured liver, and these changes can be attenuated by Sal. Furthermore, ERK and p38 inhibition blocked the decrease observed in the CI following Cd treatment (Fig 4C and 4E). These results showed that ERK and p38 play a crucial role in Cd-induced hepatotoxicity and they are in line with previous findings. However, it should be noted that Chen et al. [48] have developed a different opinion. They found that activation of p38 MAPK is not involved in Cd-induced cell death in PC12 cells, suggesting that p38 may play a different role in different cell types.

In addition, MAPKs are considered to play important roles in GJIC [47, 49]. Previous findings have shown that H_2_O_2_-induced GJIC inhibition is involved in both ERK and p38 MAPK activation [50, 51], while Cx32 plays a critical role in biological processes of hepatocyte proliferation and cell death [35]. Inhibition of ERK and p38 MAPK-attenuated oxygen–glucose deprivation has been shown to induce Cx32 up-regulation and hippocampal neuron injury [52]. Our findings show that the MAPK inhibitors recover GJIC that was inhibited by Cd (Fig 5B) and inhibition of ERK and, to some extent, p38 blocked the Cd-induced down-regulation of Cx32 mRNA expression (Fig 5A). In the co-culture system, we observed that U0126 and SB202190 had a protective effect on BRL 3A cells injured through communication with Cd-exposed cells (Fig 5C). Meanwhile, GA, a GJIC blocker, had little effect on the Cd-induced activation of ERK, JNK and p38 (Fig 5D). The results demonstrate that MAPKs induce changes in GJIC most likely by controlling connexin gene expression[53].

In summary, the present work shows that Cd induces rat hepatotoxicity via inhibition of GJIC and activation of MAPK pathways. Interestingly, inhibition of GJIC has the dual effect of protecting healthy cells, while exacerbating injury in damaged cells. ERK and p38 have been found to play critical roles in Cd-induced hepatotoxicity and mediate the function of gap junctions. Finally, Sal may be a potent chemopreventive agent that can prevent the negative effects of Cd via GJIC and MAPK pathways.
